# *“I don’t let anybody die on my watch”*: perspectives on the intersection of community overdose response and emergency medical services among people who use drugs in Seattle, WA

**DOI:** 10.1186/s12954-025-01193-0

**Published:** 2025-03-28

**Authors:** Courteney Wettemann, David L. Perlmutter, Tessa Frohe, Taylor Ryan, Grover “Will” Williams, Nathan Holland, Rachel Rourke, Robert Pitcher, Callan Elswick Fockele, Brenda Goh, Germaine Billingsley, Jenna van Draanen

**Affiliations:** 1 Department of Child, Family, and Population Health Nursing, University of Washington, Seattle, WA USA; 2 Department of Health Systems and Population Health, University of Washington, Seattle, WA USA; 3 Research with Expert Advisors on Drug Use (READU), Seattle, WA USA; 4 Department of Psychiatry and Behavioral Sciences, University of Washington, Seattle, WA USA; 5 Department of Emergency Medicine, University of Washington, Seattle, WA USA; 6 Present Address: University of Washington, Department of Health Systems and Population Health, Hans Rosling Center for Population Health, 3980 15th Avenue NE, Box 351616, Seattle, WA 98195-1616 USA

**Keywords:** People who use drugs, Harm reduction, Overdose response, Emergency medical services, Naloxone

## Abstract

**Background:**

The increasing implementation of harm reduction strategies such as take-home naloxone has placed people who use drugs (PWUD) in the position of overdose responders during overdose events, but the perspectives of PWUD are underrepresented in public health policy and practice concerning overdose response. We conducted this study to examine PWUD’s perspectives on first response services for overdose and to learn how PWUD can be supported more effectively when they respond to overdoses.

**Methods:**

The Research with Expert Advisors on Drug Use (READU) team, a community-based research team that includes academically trained researchers and people with lived and living experience conducted 13 semistructured interviews with PWUD in King County. The data were analyzed via thematic analysis. The Consolidated Framework for Implementation Research (CFIR) was used to guide the development of the interview protocol and as a framework for qualitative codebook development.

**Results:**

Participants were asked to describe their experiences with EMS, including police, during overdose response. Most had reversed an overdose themselves and demonstrated commitment to their role as overdose responders. Participants had mixed feelings about EMS involvement in overdose response, citing concerns about stigma and coercion. Police response was described as negatively impacting peer overdose response, with participants stating that past experiences of arrest and harassment by police during overdose response contributed to their reluctance to call 911 during an overdose.

**Conclusion:**

The findings from this study demonstrate the important role of PWUD in overdose response and suggest that improving interactions between EMS and PWUD could positively impact future responses, including increasing PWUD’s willingness to call 911 during overdose events.

## Background

As overdose persists as a leading cause of death among adults in the United States [1], people who use drugs (PWUD) are frequently able to recognize an overdose and administer naloxone [2]. Take-home naloxone (THN) programs provide PWUD with tools and knowledge to recognize and reverse an overdose and are effective at reducing overdose mortality [3, 4]. PWUD are well-positioned as peer overdose responders because of their immediate proximity to people who are at risk of overdose [5–7]. Emergency medical services (EMS) may take several minutes or longer to arrive [8]. Thus, equipping bystanders can result in faster administration of naloxone. In the context of this paper, EMS refers to all formal first responders, including firefighters, paramedics, and police. PWUD who respond to overdose in an unpaid community-based context are referred to in this paper as peer overdose responders.

Despite the frequency with which PWUD serve as peer overdose responders, they are under supported in this role [9]. There is substantial research devoted to understanding the impact of frequent overdose responses on EMS [10, 11], as well as institutional investment in interventions designed to reduce job-related stressors, burnout, and stigma directed toward PWUD [12, 13]. However, a commensurate understanding of the experiences of peer overdose responders is lacking.

Furthermore, EMS response to overdose provides an opportunity for linking PWUD to appropriate medical and ancillary services for treatment of other medical issues and for connection to substance use or other behavioral health services [14, 15]. However, PWUD cite fear of arrest, harassment, having their belongings taken from them, and other negative consequences as reasons not to contact EMS after an overdose [16–19]. As such, there is a need to understand how to make post-overdose care acceptable to PWUD.

The present study was conducted in Seattle, WA, where overdose mortality rates increased significantly in the two years preceding data collection. Between 2020 and 2023, the number of overdose deaths more than doubled in King County, with most occurring in the Seattle area [20]. The study also followed a major drug policy shift. In 2021, the Supreme Court of the U.S. state of Washington overturned a major drug possession law, resulting in a temporary decriminalization of simple drug possession [21]. This introduced additional uncertainty concerning the involvement of EMS in overdose response in a context where there was already confusion. The state passed a Good Samaritan Law more than a decade prior which provides some immunity from criminal prosecution for overdose survivors and bystanders who seek medical care [22], but knowledge of the law was low among a sample of paramedics and police, with the latter group often misunderstanding the sorts of charges the law provided immunity for [23].

This type of interplay between policy changes and inter- and intrapersonal dynamics underscores the complexity of real-world implementation of overdose response interventions. The Consolidated Framework for Implementation Research provides a coherent set of constructs, situated within five domains, to study program implementation [24]. The interviews with EMS and PWUD conducted concurrently in this study used this framework to study the implementation of evidence-based interventions in EMS settings [19, 25, 26]. These findings described some of the barriers and facilitators to implementing take-home naloxone, field-based buprenorphine, and field-based HIV and hepatitis C screening. The present study builds on this work not only to describe the priorities, preferences, and needs of potential post-overdose intervention recipients but to also to characterize parallel networks of peer overdose response. These networks are a component of the implementation context and provide useful insight concerning the demand for and feasibility of post-overdose service innovation. In other words, what can the expertise of PWUD, who already shoulder a considerable burden of overdose response work, teach us about how to tailor EMS-delivered overdose response and prevention innovations? We aim to answer this question by examining PWUD’s perceptions of their role as peer responders via qualitative methods designed and implemented by a community-based collaborative research team.

## Methods

### Community engagement

Each phase of this study was conducted collaboratively by an interdisciplinary team of researchers, including people with lived and living experience using drugs and formally trained researchers. The Research with Expert Advisors on Drug Use (READU) team met weekly to collaborate on the study design, interview guide drafting, sampling, data collection, data analysis, and results dissemination.

### Data collection

This study was approved by the University of Washington Institutional Review Board (#STUDY00014039). The data for this study were collected from May 2022 through June 2022. The participants were recruited and interviewed at three locations in Seattle, Washington, USA. These sites included two drop-in syringe service programs and a mobile medical provider site. The inclusion criteria for the interviews included: having experience with opioid use, being an English speaker, being at least 18 years of age, and having had any kind of EMS interaction within the past 12 months. A convenience sampling approach was used, wherein participants self-selected into the interviews. The interviews were conducted in person with 13 participants. Table 1 presents demographic information about the sample. The interviews lasted between 15 and 45 min, and the participants received a 50-dollar gift card as an honorarium. The participants were provided with information about the purpose of the study and the kinds of questions they would be asked and were informed that they could leave at any point and keep the gift card. The interviews were conducted in person by either one or two researchers at a time (CW, DP, TF, GW, NH, JVD). The interviewers consisted of formally trained researchers with backgrounds in qualitative methods, as well as READU researchers who received training and supervision in interviewing. A formally trained researcher was present for each interview and was joined by a READU researcher for at least half of the interviews conducted.

The interviews were audio-recorded and transcribed via a professional transcription service. The research team regularly reviewed the data for saturation at team meetings until topics arising from earlier interviews became redundant in later interviews, and the research team did not believe that further interviews would lead to additional novel information.

**Table 1:** Participant demographics

Participant demographics	*n*
***Race/ethnicity***	
Alaska Native	1
Hispanic/Latinx	2
Mixed Race	3
Prefer not to say	3
White	4
***Gender***	
Male	8
Female	2
Non binary	1
Prefer not to say	2
***Age***	
26–35	6
36–45	4
46–55	1
Prefer not to say	2
***Years Using Substances***	
< 1	1
3–9	5
10–14	3
> 15	1
Prefer not to say	3

### Data analysis

Thematic analysis was used, with a hybrid inductive-deductive approach to coding [27]. The data analysis began with all the researchers reviewing the transcripts to familiarize themselves with the data. Transcripts were annotated to build an initial set of impressions from the transcript. The team then selected a subset of relevant constructs from CFIR as an initial guide for organizing impressions categorically, focused on constructs within the *outer setting domain; the inner setting domain;* and *individuals (roles and characteristics) domain*. With impressions clustered together under relevant constructs, the team discussed and then labeled the unique ideas in each cluster. This constituted an initial codebook. Two coders independently read each transcript and applied this set of codes. The group then reviewed the first coding session to reconcile inconsistencies in the coding process and update the codebook for definitional clarity. The dataset was subsequently coded once more by two independent coders. Once all the transcripts were coded, the research team met to review coding patterns across the dataset and organize them into themes, which were formally defined and named. While the CFIR was used for conceptual clarity and organization, the team chose not to present CFIR domain and construct notation in the results in favor of using participant-chosen language about the *settings* and *individual roles and characteristics.* (see Table 2).

## Results

**Table 2:** Summary of themes

Theme	Description
PWUD as peer overdose responders	Perceived role in overdose response and impressions of overdose response services rendered by EMS. Participants broadly described significant experience and personal investment in responding to overdose, citing a strong sense of personal responsibility and confidence in overdose response skills, although some participants reported negative emotional impacts from frequently witnessing overdoses.
PWUD role in emergency medical services response	As frequent bystanders, participants said they felt responsible for making decisions around EMS involvement in overdose response, weighing the benefits with potential consequences (fear of negative repercussions, exposure to stigma), and noting that overdoses are often successfully reversed by bystanders without EMS.
Fear of police response among peer overdose responders	Police response was a primary factor discouraging PWUD from calling EMS during an overdose. Participants broadly perceived police overdose response as unhelpful and unsafe.

The participants in this study described their perceived role in overdose response and their impressions of the overdose response services rendered by emergency medical services, including police. The participants broadly articulated significant experience and personal investment in responding to overdose, citing a strong sense of civic duty and confidence in their ability to respond, although some participants reported negative emotional impacts from frequently witnessing overdoses.

As frequent bystanders, participants said they felt responsible for making decisions around EMS involvement in overdose response, weighing the benefits with potential consequences (fear of negative repercussions, exposure to stigma), and noting that overdoses are often successfully reversed by bystanders without EMS.

Lastly, police response was a primary factor discouraging PWUD from calling EMS during an overdose. The participants broadly perceived police overdose response as unhelpful and unsafe.

### PWUD as peer overdose responders

The participants in the study repeatedly emphasized the importance of peers in overdose response. PWUD’s personal investment in the well-being of their peers was demonstrated here through how frequently they reversed overdoses and the sense of duty they felt to do so. Their practical readiness and consistency in responding highlight how seriously the participants took this role.

Almost all the participants reported being familiar with overdose response protocols and knowledgeable about best practices for overdose reversal with naloxone, including how to provide rescue breathing. Most participants said they always carry naloxone with them, not for their own safety (as one participant put it, *“you can’t Narcan yourself”*), but out of care for others. Several participants said that they expected other people in their community to carry it as well. As one participant stated:

I don’t think you could ever have enough of that stuff. ‘Cause you never know. Maybe it’s not you, it could be somebody walking down the street, you know, you never know. -Interview 10.

The readiness to respond to overdose was linked to several factors, including the availability of naloxone and the frequency of overdose. Most of the participants could describe at least one instance where they had responded to an overdose, and several had responded multiple times.

Feelings of personal responsibility also emerged as a factor, as the quote below illustrates:

When I was in a tent, when I lived in a tent, I saved somebody literally every day. Every day. I just don’t let anybody die around me… I always carry Narcan, so I would make sure that I revive them, I do the proper procedures… I don’t let anybody die on my watch, so I just tell people, if you’re gonna do drugs, have that care for your well-being around you versus somebody you don’t know. -Interview 1.

The participant’s investment in being a part of overdose response extended beyond overdose reversal. The participants described taking on other crucial roles, including naloxone distribution within their social groups, advising peers on safer practices for using drugs, and keeping an eye on others while they used. The participants in this study often reported using substances in encampments or other congregate settings, where a person experiencing overdose may be more visible to others and peers nearby can administer aid swiftly.

A sense of civic duty was present throughout the interviews; participants noted that they are often the first to respond to overdose and are sometimes the only responders, as illustrated by this interviewee:

Most of the time when I see an overdose there isn’t [an EMS response]… It’s people like me on the street that have trained themselves in medical. And honestly, I know a lot now I’ve taught myself so I could help people. But most of the time it’s us before the paramedics even get there, if it ain’t us, then they’re usually dead. -Interview 13.

The participants described mixed feelings during and after the event, regardless of how many times they had responded. The participants reported that positive feelings such as pride and a sense of accomplishment were common after they revived someone from an overdose, alongside feelings of stress, concern, and panic. Multiple participants described cumulative feelings of distress after repeatedly witnessing overdoses. The participants reflected on memories of past overdose events where other peers were present and said they perceived anxiety in them as well, especially among those not accustomed to witnessing overdoses. Despite this, the participants consistently described overdose response as a routine, almost mundane task: something to expect and be prepared for.

### PWUD considerations about engaging with EMS

The participants emphasized their role as decision makers during overdose events and described the factors they considered when deciding whether to call 911, including their impressions of past experiences with EMS.

Citing the prevalence of THN and their own competence in using it, participants said they felt empowered to respond to overdose themselves. Most participants said they expected many overdoses to be reversed without EMS involvement. Factors that were cited as motivating participants to call EMS included the absence of naloxone or the failure of initial doses of naloxone to revive someone. The participants reported being deterred from contacting EMS when they feared arrest or had more experience reversing overdoses. In the quote below, a participant describes the circumstances under which they would call 911:

Interviewer: So you don’t feel like you’d call 911 if you had Narcan?

Interviewee: No.

Interviewer: Do you feel like you would call 911 if you didn’t have Narcan?

Interviewee: […] If you’re not breathing and you’re not responding and you’re not coherent, something is wrong, and if I don’t know CPR, I’m definitely calling 911. -Interview 2.

Peer response was preferred over EMS involvement because of participants’ experiences with stigmatization and criminalization during EMS interactions. The participants also noted that once an EMS responder was involved, the situation was likely to be lifted from peer control to the EMS responder. The preferences of the person who overdosed might be known and considered by peers present at the scene; some participants described reluctance to call not only because they were concerned about being arrested themselves, but also because of respect for the wishes of the person who overdosed.

Avoidance of the emergency department (ED) also played a role in the decision to call 911. The participants said that they viewed ED services post-overdose as unhelpful, stigmatizing, and painful, citing long waits and few practical resources offered. To accept transport to the ED, participants described being forced to leave their pets behind, as well as their belongings and shelters, if they were living outside, leaving them vulnerable to theft or harm.

The participants also reported positive EMS interactions. Positive interactions with EMS increased the willingness to call 911 for some participants, as the quote below illustrates:

That’s only happened to me once [calling EMS during an overdose]. And it was not me, my friend overdosed, and it was actually decent…Everyone’s always afraid you’re gonna go to jail when you call about someone overdosing, you know, since they changed that. And that makes a huge difference ‘cause people are actually willing to call when someone’s hurt. -Interview 10.

The participants who were satisfied with their interactions with EMS described the EMS responder’s behavior as empathetic, receptive, and respectful of the autonomy of the person who overdosed. Providing clear instructions and keeping other bystanders calm at the scene of an overdose were noted and appreciated by the participants. Ensuring that the person who overdosed was as comfortable as possible and given space, instead of trying to force an outcome preferred by EMS, was viewed positively by the participants.

The participants’ negative interactions with EMS were characterized by stigmatizing language, coercion, and perceived invasive questioning of other PWUD at the scene. They also described frustration with not feeling listened to by EMS after initiating an overdose response. Following these interactions, the participants said they were discouraged from calling 911. Some participants perceived positive changes in these interactions over time and speculated that EMS could have received training that made them more collaborative and less stigmatizing during overdose response.

Participants in this study demonstrated commitment to responding to overdose even when they chose not to call 911. Those who said they had declined to call in the past were not motivated by a lack of care but by their faith in their own competency to respond and their desire to avoid potential negative consequences and stigma during EMS response-both for themselves and for the person who overdosed.

### Fear of police response among peer overdose responders

The type of EMS responder that participants expected to interact with impacted their willingness to call. While the participants in this study held mixed feelings about firefighters and paramedics, their views of police were largely negative. Several participants stated that the primary motive of police at the scene of an overdose was punitive action instead of support, describing police attempting to interrogate people about their drug use before they had recovered from their overdose or treating them roughly, which some participants saw as an attempt to punish people extrajudicially for their drug use. As one participant described,

I was in shock. ‘Cause the EMTs were fine but the cop… He was helping on getting the guy’s address, and the guy […] was barely able to contemplate breathing. -Interview 6.

The participants said that the risk of arrest discouraged them from calling EMS and stated their belief that other PWUD felt the same. In fact, some participants felt that police responses to overdoses made responses from PWUD less effective because they would not feel safe staying at the scene to monitor respiratory distress and administer aid. The quote below highlights one participant’s perspective:

A lot of people would be like, ‘Oh my God, the cops are coming. We gotta go. We gotta go.’ And they start freaking out. So, it’s just like once the EMT comes and the firefighters, they feel comfortable. Now I feel, if it was more police officer presence, it would be more deaths…Cause people get scared, they’ve got warrants and they don’t wanna to go to jail. -Interview 1.

While participants noted punitive behaviors taken by every type of EMS responder, they most heavily associated them with the police. Some participants felt that police officers were primarily interested in responding to overdose to administer punishment. Multiple participants cited the perceived reluctance of police officers to respond to overdoses following the temporary decriminalization of drug possession in the area, even when they were already present at the scene. The participants said that they preferred police to respond and aid if they were already present, but preferred police call in other EMS to provide any care beyond naloxone administration and rescue breaths.

## Discussion

The PWUD interviewed in this study showed a firm sense of responsibility to be prepared to respond to overdose at any time. This sense of responsibility, and the belief among PWUD that it is important to carry naloxone owing to the likelihood of witnessing an overdose, is well represented in prior research [28, 29]. In addition to being prepared to administer naloxone, the participants described several other functions of their role as peer overdose responders, namely distributing naloxone to their social networks, advising others on strategies to reduce overdose risk, and supervising others’ use. This highlights the importance of social support and networks, which have been shown to be protective factors in overdose prevention [30]. Elkhalifa and colleagues’ peer researcher-led study of PWUD in British Columbia, Canada described how social networks and relationships among PWUD reduce harm among peers– through the provision of emotional support (e.g., asking someone how they are doing), tangible support (e.g., lending money, providing harm reduction supplies), and informational support (e.g., showing someone how to use naloxone) [31]. Our findings contribute to this line of inquiry by adding to the understanding of the personal motivation for some individuals to contribute to reducing overdose risk and other harms in their social networks. Many of the participants we interviewed characterized carrying naloxone as an obvious choice; it is typical for them to expect to witness an overdose, and administering naloxone oneself is quicker and considered safer than waiting for EMS to arrive.

Despite being prepared to administer naloxone, some participants described feelings of stress, concern, and panic while responding to an overdose and even reported a lasting negative emotional impact. PWUD interviewed by Wagner and colleagues (2014) also described how peer overdose responders experienced feelings of regret for not being able to save others in the past, felt burdened by being the person to whom others come during an emergency, and developed strained relationships with individuals whom they have revived on multiple occasions [32]. Moreover, witnessing an overdose is associated with symptoms consistent with posttraumatic stress disorder [33]. This points to the need for follow-up services available not only to the survivor of an overdose but also to peer overdose responders and other bystanders. Increased motivation for behavior change may follow witnessing an overdose [34], as well as heightened risk associated with substance use or mental health symptoms [35]. The period immediately following an overdose should be considered a crucial window for intervention, whether to offer substance use-related services or provide care for the emotional and psychological toll of overdose response. Prior studies have identified a small number of EMS co-response programs that offer referrals and post-overdose peer support to overdose survivors and their social networks, including bystanders, but more research is needed to examine the impact of these interventions on peer overdose responders [36, 37].

Promoting appropriate utilization of EMS during or after an overdose is important owing to the elevated risk for subsequent overdose when individuals are not transported to the hospital [35] as well as the risk of medical complications that naloxone will not address [14]. A potential barrier to providing post-overdose care was noted by participants in this study through a common reticence to contact emergency services. The anticipation of inappropriate or harmful treatment by EMS was a principal factor in deciding whether to call 911 for nearly all of the participants in this study. A lack of perceived relevance of services and confidence in their ability to reverse the overdose themselves were also cited as reasons for not contacting 911. The participants described varied experiences with EMS, but instances of mistreatment were reported by almost all the participants in this study. The harmful effects of stigmatization by EMS have similarly been detailed by PWUD in previous research [38, 39] and are echoed by measures of stigma among EMS and other healthcare providers [39]. PWUD perceive a delegitimization and devaluing of their labor and expertise by EMS, contributing to a feeling of antagonism [35]. Some participants also held positive feelings toward EMS and praised them for their efficiency and care during overdose response. The participants felt that their hesitancy to contact 911 could be reduced if EMS were more consistently collaborative and respectful during overdose response. Tailored training focused on improving EMS attitudes towards PWUD has shown some promising results, particularly when training content is co-delivered by overdose survivors, but more research is needed to learn whether self-reported improvements in attitudes among EMS towards PWUD translate to improved interactions during overdose response [40].

Distinct from EMS involvement were participants’ perceptions concerning police presence at overdoses. The participants unanimously opposed police involvement in overdose response, apart from basic naloxone administration and basic aid if police were already present at the scene. Awareness of police power to arrest or take other punitive action against people at the scene negated the potential for building trust with participants. This finding is consistent with a recent scoping review of barriers to calling for emergency services, which identified concerns about police presence as the primary barrier across 48 studies [41]. Research exploring police officers’ own perceptions of their place in the overdose crisis suggests that they themselves hold the perception that their current role is ineffectual, leading to professional role strain as well as tension with communities [42–45]. Despite the fact that this study took place in a state with Good Samaritan Laws which afford some immunity from criminal prosecution when calling for medical attention during an overdose, anticipation of arrest or another harmful police encounter persisted, a finding that is consistent with other research on barriers to contacting EMS when such laws are in place [16, 46]. Our findings and those of several other studies suggest that by involving police in most overdose response calls, there is a risk of affecting the appropriate utilization of emergency services and complicating the implementation of post-overdose interventions.

There is a growing body of literature on the promise of EMS-delivered interventions for PWUD, but polarized opinions among EMS concerning PWUD and certain kinds of harm reduction interventions could complicate implementation [47]. Some limited examples of overdose-related interventions developed collaboratively with PWUD and EMS partners show promise in designing interventions that meet the needs of all parties [48]. Despite this, EMS-delivered interventions that are tailored to the needs of PWUD remain rare. Champagne-Langabeer and colleagues identified only 27 such programs across nearly 22,000 EMS agencies in the United States [49]. Among those 27, most provided access to some kind of harm reduction supplies, such as naloxone, or referrals to specialists to support overdose survivors, but fewer included a peer support component or facilitated linkage to medication for opioid use disorder treatment [49]. Evidence from specialized post-overdose outreach programs indicates that EMS-delivered post-overdose interventions are effective at linkage to and retention in treatment [50]. Rather than offering access to a limited suite of harm reduction services or treatment, some models of post-overdose care aim to facilitate access to a wider set of resources, depending on an individual’s preferences. New York State’s Post-Overdose Response Teams provide timely linkage to medications for opioid use disorder but also social services, primary or mental health care, and assistance making or getting to other kinds of appointments, with an outreach team composed of people with lived experience of substance use [51]. As our study has shown, many PWUD already feel capable and equipped with the tools needed to respond to overdoses. This raises the question of which services PWUD would be most desirous of in the time following an overdose, and of those which are appropriate and feasible to deliver via EMS or similar channels. Compensated opportunities for PWUD to participate in co-designing post-overdose interventions could be useful for identifying these areas of improvement in post-overdose care. Findings from this study also suggest that centering the experiential knowledge of PWUD and recognizing their critical contributions to overdose response within EMS systems could lead to more positive relationships between PWUD and EMS.

### Limitations

Our study used a small, convenience sample, thereby limiting generalizability. It was conducted in an area where harm reduction services operate legally and are relatively well resourced. All participants were recruited from the same harm reduction sites where they have access to THN, overdose response training, and other resources. Their experiences may differ from those of PWUD who face more barriers to these resources or who live in different social or geographical conditions.

## Conclusion

Our findings underscore the way in which the proliferation of naloxone throughout communities has been successful as an accessible means of reducing mortality. That said, we have also identified ways in which this shifting of responsibility to PWUD has not been accompanied by a commensurate transformation in the care available to overdose survivors and those peer overdose responders who save their lives. These findings point to unmet needs among peer overdose responders, in terms of the psychological and behavioral impacts of witnessing an overdose. These are opportunities for innovations in the way people affected by the overdose crisis access timely, appropriate care. Future research could focus on identifying what services are most desired by overdose survivors and peer overdose responders after an overdose. We have also noted some potential challenges in implementing such services. Our findings also suggest that overdose response can be improved through incorporating strategies that prioritize positive relationships and collaboration between EMS and PWUD, making services that PWUD perceive as more appealing and relevant available alongside EMS and reducing unnecessary police contact.

## Data Availability

The datasets used and/or analyzed during the current study are available from the corresponding author on reasonable request.
